# Rhizoma Coptidis: A Potential Cardiovascular Protective Agent

**DOI:** 10.3389/fphar.2016.00362

**Published:** 2016-10-07

**Authors:** Hui-Li Tan, Kok-Gan Chan, Priyia Pusparajah, Acharaporn Duangjai, Surasak Saokaew, Tahir Mehmood Khan, Learn-Han Lee, Bey-Hing Goh

**Affiliations:** ^1^Novel Bacteria and Drug Discovery Research Group, School of Pharmacy, Monash University MalaysiaBandar Sunway, Malaysia; ^2^Biomedical Research Laboratory, Jeffrey Cheah School of Medicine and Health Sciences, Monash University MalaysiaBandar Sunway, Malaysia; ^3^Division of Genetic and Molecular Biology, Faculty of Science, Institute of Biological Sciences, University of MalayaKuala Lumpur, Malaysia; ^4^Center of Health Outcomes Research and Therapeutic Safety, School of Pharmaceutical Sciences, University of PhayaoPhayao, Thailand; ^5^Division of Physiology, School of Medical Sciences, University of PhayaoPhayao, Thailand; ^6^Faculty of Pharmaceutical Sciences, Pharmaceutical Outcomes Research Center, Naresuan UniversityPhitsanulok, Thailand; ^7^Department of Pharmacy, Abasyn University PeshawarPeshawar, Pakistan

**Keywords:** coptis root, Huang Lian, *Coptis chinensis* Franch, cardiovascular diseases, ethnopharmacology

## Abstract

Cardiovascular diseases (CVDs) are among the leading causes of morbidity and mortality in both the developed and developing world. Rhizoma coptidis (RC), known as Huang Lian in China, is the dried rhizome of medicinal plants from the family Ranunculaceae, such as *Coptis chinensis* Franch, *C. deltoidea* C.Y. Cheng et Hsiao, and *C. teeta* Wall which has been used by Chinese medicinal physicians for more than 2000 years. In China, RC is a common component in traditional medicines used to treat CVD associated problems including obesity, diabetes mellitus, hyperlipidemia, hyperglycemia and disorders of lipid metabolism. In recent years, numerous scientific studies have sought to investigate the biological properties of RC to provide scientific evidence for its traditional medical uses. RC has been found to exert significant beneficial effects on major risk factors for CVDs including anti-atherosclerotic effect, lipid-lowering effect, anti-obesity effect and anti-hepatic steatosis effect. It also has myocardioprotective effect as it provides protection from myocardial ischemia-reperfusion injury. These properties have been attributed to the presence of bioactive compounds contained in RC such as berberine, coptisine, palmatine, epiberberine, jatrorrhizine, and magnoflorine; all of which have been demonstrated to have cardioprotective effects on the various parameters contributing to the occurrence of CVD through a variety of pathways. The evidence available in the published literature indicates that RC is a herb with tremendous potential to reduce the risks of CVDs, and this review aims to summarize the cardioprotective properties of RC with reference to the published literature which overall indicates that RC is a herb with remarkable potential to reduce the risks and damage caused by CVDs.

## Introduction

Cardiovascular diseases (CVDs) appears set to continue as the largest cause of death and disease burden across the globe. They include a wide spectrum of life-threatening disorders such as coronary heart disease (CHD), cerebrovascular disease and peripheral arterial disease, all of which result from impairment to the heart and blood vessels (Wallace, 2011). Among the risk factors strongly associated with these disorders are high levels of low-density lipoprotein (LDL) cholesterol, hypertension, diabetes, and abdominal obesity (Walden and Tomlinson, 2011).

**Figure 1:**
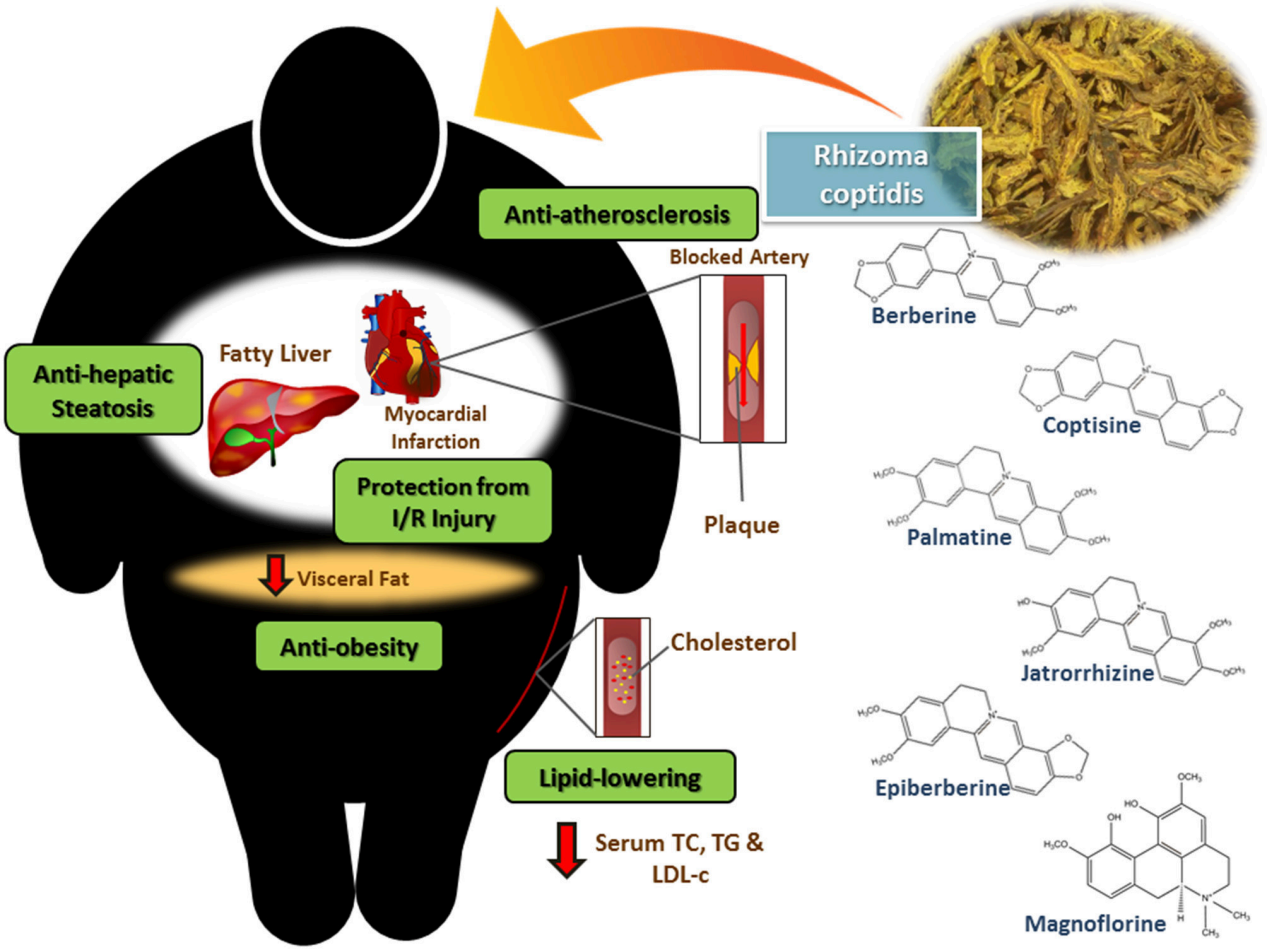
**Rhizoma coptidis which contains alkaloids such as berberine, coptisine, palmatine, epiberberine, jatrorrhizine and magnoflorine exerts cardioprotective activity through its anti-atherosclerotic effect, protection from myocardial ischemia-reperfusion injury, lipid-lowering effect, anti-obesity effect and anti-hepatic steatosis effect**.

Rhizoma coptidis (RC), known as Huang Lian in China, is the dried rhizome of medicinal plants from the family Ranunculaceae, including *Coptis chinensis* Franch, *C. deltoidea* C.Y. Cheng et Hsiao, and *C. teeta* Wall (Chen et al., 2008; Ma et al., 2012). It is a well-known herb in traditional Chinese medicine and has a long history with its pharmacological uses first mentioned in the Shen Nong Ben Cao Jing (a compilation of information regarding Chinese herbs dating back to 2800 BC) in the Eastern Han Dynasty (Yi et al., 2013). Ancient beliefs state that it is “cold” in nature and is able to remove damp heat, fire or toxicity (Wang et al., 2014). For over 2000 years, Chinese medicinal physicians have used RC as a food additive and herbal medicine for its antibacterial, antiviral, anti-inflammatory, anti-hyperglycemic and hypolipidemic activities (Kou et al., 2016). Today, RC is still widely utilized in herbal medicine for the treatment of various conditions. This is evident based on a survey of patented medicines in China which reveals that RC is commonly used as one of the ingredients in preparations to treat obesity, diabetes mellitus, hyperlipidemia, hyperglycemia and lipid metabolism disorders (Chen, 2009; Ye et al., 2009; Guo, 2012; Li et al., 2015; Wang, 2015). Given the potential benefits in seeking new approaches to treating and preventing CVDs, there has been tremendous interest among the scientific community in exploring the biological properties of RC and providing scientific evidence for its traditional medical uses. At the same time, it is also crucial to investigate the phytoconstituents that are responsible for the biological properties (Moghadamtousi et al., 2013; Tan et al., 2015). Based on current knowledge, the major bioactive compounds contributing to RC's bioactive properties are berberine, coptisine, palmatine, epiberberine, jatorrhizine and magnoflorine, as illustrated in Figure 1 (Hung et al., 2007a; Kou et al., 2016). There is a large of body of work suggesting that RC has protective properties against several major risk factors and damage caused by CVDs. This review aims to summarize the currently available evidence of RC's cardioprotective properties—both *in vitro* and *in vivo* studies were included and are summarized in Table 1.

**Table 1:** **Summary of cardioprotective activity of Rhizoma coptidis**.

**Effect**	**Extract/compound (s)**	**Experiment**	**Effect**	**Possible mechanism**	**References**
Anti-atherosclerotic effect	Berberine	*In vitro*	Inhibition of foam cell formation and reduced cholesterol accumulation in macrophages. Synergistic effect with atorvastatin in preventing atherosclerotic processes	Activation of AMPK-SIRT1-PPARγ pathway, decreased uptake of ox-LDL	Chi et al., 2014
	Ger-Gen-Chyn-Lian-Tang (Puerariae radix, scutellariae radix, coptidis -rhizoma and glycyrrhizae radix)	*In vitro* and *in vivo*	Decreased serum levels of TC, LDL, atherosclerotic lesions and collagen expression in atheroma plaques. Decreased VSMCs migration and lipid accumulation in hepatocytes	Activation of AMPK signaling	Ho et al., 2012
	Berberine	*In vitro*	Inhibition of generation of ROS and subsequent mitochondrial membrane potential collapse, chromosome condensation, cytochrome C release, and caspase-3 activation induced by ox-LDL in HUVECs	Suppression of ROS overproduction, through modulation of expression or activity of ROS generating enzyme and ROS scavenging enzyme	Hsieh et al., 2007
	Berberine	*In vitro*	Reduced expression of MMP-9 and EMMPRIN in PMA-induced macrophages	Suppression of activation of p38 pathway	Huang et al., 2011
	Magnoflorine	*In vitro*	Inhibitory effect against Cu2+-induced lipid peroxidation of HDL and inhibition of generation of TBARS	Antioxidant action of magnoflorine	Hung et al., 2007a
	Magnoflorine	*In vitro*	Inhibitory effect against Cu2+-induced lipid peroxidation of LDL, glycated LDL and glycoxidated LDL. Inhibition of generation of TBARS	Antioxidant action of magnoflorine	Hung et al., 2007b
	Rhizoma coptidis extract and berberine	*In vitro*	Inhibition of LPS-induced MCP-1 production in murine macrophages	Inhibited activation of the transcription factors AP-1 and NFκB	Remppis et al., 2010
	Berberine	*In vitro* and *in vivo*	Reduced aortic lesions, oxidative stress and expression of adhesion molecules in aorta of ApoE−/− mice. Increased UCP2 mRNA and protein expression in HUVECs	AMPK-dependent upregulation of UCP2 expression	Wang et al., 2011
	Coptisine	*In vitro* and *in vivo*	Inhibition of LPS stimulated expression of inflammation-associated genes in mouse, human macrophages and in carrageenan-induced rats paw edema	Inhibition of activation of NFκB, MAPK, and PI3K/Akt activation	Wu et al., 2016
Lipid-lowering effect	Rhizoma coptidis alkaloids extract	*In vivo*	Decreased serum TC, TG, LDL-c, increased bile acid level in the liver of hyperlipidemic rats	Increased cholesterol conversion into bile acids by up-regulating gene expression of CYP7A1, increased its activity in the liver due to positive regulation of PPARα and negative modulation of FXR	Cao et al., 2012
	Coptisine	*In vitro*	Decreased TC and LDL-c in HepG2 cells	Up-regulated mRNA and protein expressions of LDLR and CYP7A1, down-regulated mRNA and protein expressions of HMGCR	Chen et al., 2015
	Rhizoma coptidis total alkaloids, berberine, coptisine, palmatine, epiberberine and jatrorrhizine	*In vivo*	Decreased serum TC, TG, LDL-c and increased HDL-c, TBA levels in feces of hyperlipidemic hamsters	Down-regulated expression of HMGCR and up-regulated expression of LDLR and CYP7A1 as well as promoting the excretion of TBA in the feces	He et al., 2015a
	Coptisine	*In vivo*	Decreased serum TC, TG, LDL-c, increased HDL-c, TBA levels in feces of hyperlipidemic hamsters	Inhibited cholesterol synthesis through suppressing the HMGCR expression and promoting usage and excretion of cholesterol via up-regulating LDLR and CYP7A1 expression	He et al., 2015b
	Rhizoma coptidis total alkaloids, berberine, coptisine, palmatine, epiberberine and jatrorrhizine	*In vitro* and *in vivo*	Reduced lipid and cholesterol accumulation in HepG2 cells; decreased serum TC, TG, LDL-c and increased HDL-c, TC, TBA levels in feces of hypercholesterolemic hamsters	Up-regulation of LDL receptor and CYP7A1, as well as HMGCR downregulation	Kou et al., 2016
	JiuHuangLian (Rhizoma coptidis steamed with rice wine)	*In vivo*	Decreased serum TC, TG, and free fatty acid levels in diabetic rats	Regulatory effect on glucose-lipid metabolism	Li et al., 2013
	Berberine, coptisine, palmatine, epiberberine and Jatrorrhizine	*In vivo*	Decreased serum TC, TG by palmatine and jatrorrhizine, increased HDL-c by berberine, palmatine and jatrorrhizine in diabetic mice	–	Ma et al., 2016
	Palmatine	*In vivo*	Decreased serum TC, TG, LDL-c, increased TC and TBA levels in feces of hyperlipidemic hamsters	Up-regulated LDLR and CYP7A1 mRNA and protein expression, down-regulated ASBT mRNA and protein expression, enhanced fecal excretion of TC and TBA	Ning et al., 2015
	Berberine	*In vivo*	Decreased serum TC, TG, LDL-c, increased HDL-c and NO level in diabetic rats	–	Tang et al., 2006
	Jatrorrhizine	*In vivo*	Decreased serum TC, TG, LDL-c, increased HDL-c and TBA levels in feces of hamsters	Improved utilization and excretion of cholesterol by up-regulating mRNA and protein expression of LDLR and CYP7A1	Wu et al., 2014a
	Berberine, coptisine, palmatine, epiberberine and Jatrorrhizine	*In vitro* and *in vivo*	Decreased serum TC, TG, LDL-c, increased HDL-c in hamsters. Increased mRNA expression of LDLR in liver and HepG2 cells	Up-regulated LDLR mRNA expression	Wu et al., 2014c
	Rhizoma coptidis extract	*In vivo*	Decreased serum TC, LDL and oxidized LDL in rats	Reduction of cholesterol synthesis	Yokozawa et al., 2003
	Berberine, jatrorrhizine, columbamine, berberrubine and demethyleneberberine	*In vitro*	Inhibition of lipid accumulation in Hep G2 cells	Up-regulated LDLR mRNA and protein expression	Zhou et al., 2014
	Epiberberine	*In vivo*	Decreased serum TC, LDL-c, TBA, increased TC and TBA levels in feces of hyperlipidemic hamsters	Inhibited cholesterol synthesis through suppressing the HMGCR expression and promoting usage and excretion of cholesterol via up-regulating LDLR and CYP7A1 expression	Zou et al., 2016
Anti-obesity	Rhizoma coptidis methanol extract, berberine, epiberberine, coptisine, palmatine, and magnoflorine	*In vitro*	Reduction of intracellular triglyceride contents and lipid accumulation in 3T3-L1 cells	Down-regulated expression of major adipogenic transcription activator (PPARγ and C/EBP- α) proteins of the adipogenesis pathway	Choi et al., 2014
	Rhizoma coptidis total alkaloids, coptisine, berberine and palmatine	*In vivo*	Reduced body weight gain, TC, TG, LDL-C, TBA and lipopolysaccharide, liver fat deposition and epididymal adipose cell size in hyperlipidemic mice. Increased abundance of *Sporobacter termitidis, Alcaligenes faecalis, Akkermansia muciniphila* in the gut of mice, decreased abundance of *Escherichia coli, Desulfovibrio C21 _c20, Parabacteroides distasonis*	Modulation of gut microbiota and lipid metabolism by acting as agonists of FXR and TGR5, activators for SREBP2, LDLR, UCP2 and CYP7A1, inhibitors of HMGCR, TXNIP, TLR4 and JNK	He et al., 2016
	Berberine	*In vitro*	Inhibition of differentiation and mitotic clonal expansion of 3T3-L1 preadipocytes	Inhibition of mRNA and protein expression of adipogenesis related transcription factors PPARγ and C/EBPα and their upstream regulator, C/EBPβ	Huang et al., 2006
	Rhizoma coptidis ethanol extract, berberine	*In vitro* and *in vivo*	Reduced body visceral adipose weights, lipid levels, degradation of dietary polysaccharides, proportions of fecal Firmicutes and Bacteroidetes to total bacteria in high-fat diet-fed mice; inhibited growth of *Lactobacillus* under anaerobic conditions	Decreased degradation of dietary polysaccharides, lowered potential calorie intake, activation of Fiaf protein and related gene (AMPK, PCG1α, UCP2, CPT1α and Hadhb) expressions of mitochondrial energy metabolism in visceral adipose tissues	Xie et al., 2011
Anti-hepatic steatosis	Sam-Hwang-Sa-Sim-Tang (Rhizoma coptidis, Scutellariae radix and Rhei rhizoma)	*In vivo*	Reduced lipid accumulation in the liver of mice	Inhibition of mRNA expression of key hepatic molecules such as SREBP2, LXR, LDLR, and HMG-CoA	Ahn et al., 2013
	Rhizoma coptidis alkaloids extract, berberine, coptisine, palmatine, epiberberine, jatrorrhizine, columbamine and magnoflorine	*In vitro*	Attenuated triglyceride accumulation in HepG2 cells	–	Fan et al., 2013
	Berberine	*In vitro* and *in vivo*	Attenuated hepatic steatosis, reduced expression of ACC and FAS in mice; reduced fat deposition in hepatocytes	Anti-inflammatory action of berberine	Guo et al., 2016
	Jiao Tai Wan (Rhizoma coptidis and Cortex cinnamomi)	*In vivo*	Attenuated hepatic lipid accumulation in diabetic rats, down-regulation of ACC and FAS protein expressions, up-regulation of AMPK and pACC protein expressions in the liver tissues; reduced TG content in patients' livers	Inhibition of lipogenic gene expression in the liver	Huang et al., 2013
	Berberine	*In vivo*	Attenuated liver steatosis in high-fat diet-induced steatotic rats	Reversal of alteration of hepatic gene expression that occurred in steatotic liver	Yuan et al., 2015
	Berberine	*In vitro* and *in vivo*	Attenuated liver steatosis in mice and decreased free fatty acid-induced lipid accumulation in cultured hepatocytes	Reduction of endoplasmic reticulum stress	Zhang et al., 2016
Protection from Myocardial Ischemia-reperfusion	Berberine	*In vivo*	Decreased infarct size, duration and incidence of arrhythmias. Reduced AMPK concentration, ratio of ADP/ATP and AMP/ATP in the myocardial risk areas; increased AMPK concentration, ratio of ADP/ATP and AMP/ATP in the non-ischemic areas.	Regulation of AMPK activity in non-ischemic and risk areas of the heart	Chang et al., 2012
	Berberine	*In vivo*	Decreased infarct size and attenuated arrhythmias. Increased AMPK activity, ratio of AMP/ATP in the non-ischemic areas	Activation of AMPK, AKT phosphorylation, and GSK3β inhibition in the non-ischemic areas of the diabetic heart	Chang et al., 2016
	Berberine	*In vitro* and *in vivo*	Improved recovery of cardiac systolic/diastolic function and reduced myocardial apoptosis in diabetic rats subjected to myocardial I/R; Reduced hypoxia/reoxygenation-induced myocardial apoptosis of neonatal rat cardiomyocytes	Activation of AMPK and PI3K–Akt–eNOS signaling	Chen et al., 2014
	Coptisine	*In vivo*	Reduced infarct size and improved cardiac function after I/R injury in rats	Suppression of myocardial apoptosis and inflammation, through inhibition of Rho/ROCK pathway	Guo et al., 2013
	Berberine	*In vitro* and *in vivo*	Enhanced H/R-induced cell viability, reduced myocardial infarct size, improved cardiac function of mouse hearts; decreased p-AMPK and p-mTORC2 in H9c2 myocytes	Inhibition of autophagy activation, through decreased expression of autophagy-related proteins such as SIRT1, BNIP3, and Beclin-1	Huang et al., 2015
	Palmatine	*In vitro* and *in vivo*	Improved I/R-induced myocardial dysfunction, inhibited increased LDH, CK and MDA contents in I/R rats serum, inhibited declined activity of SOD and catalase in I/R cardiac tissues, reduced COX-2 and iNOS expression in I/R myocardium; increased HO-1 induction in human aortic endothelial cells	Reduction of oxidative stress and modulation of inflammatory mediators	Kim et al., 2009
	San-Huang-Xie-Xin-Tang (Coptidis rhizome, Scutellariae radix and Rhei rhizome)	*In vivo*	Reduced plasma levels of cardiac enzymes, arrhythmia scores, mortality rate of rats with I/R. Reduced infarct size and apoptosis induced by I/R	Increased myocardial eNOS expression, plasma nitrite and decreased activation of ERK1/2, p38 and JNK	Liou et al., 2011
	Rhizoma coptidis extract	*In vitro* and *in vivo*	Decreased ST-T in ECG, serum levels of CK, LDH, MDA and improved SOD in rats; decreased level of LDH in neonatal rat cardiomyocytes	Improved oxidative damage of acute myocardial ischemia	Liu et al., 2010
	Berberine	*In vivo*	Decreased myocardial infarction area, decreased serum levels of CK isozyme (CK-MB), LDH and cTnl, upregulated expression of Bcl-2 and mitochondrial cytochrome c and downregulated expression of Bax and cytosolic cytochrome c	Attenuation of myocardial apoptosis and improved mitochondrial dysfunction	Wang et al., 2015
	Berberine	*In vitro* and *in vivo*	Improved cardiac function recovery, decreased myocardial apoptosis, infarct size, serum CK, LDH; attenuation of I/R-induced myocardial apoptosis of cultured cardiomyocytes	Modulation of Notch1/Hes1-PTEN/Akt signaling pathway	Yu et al., 2015
	Berberine	*In vitro* and *in vivo*	Reduced I/R-induced myocardial infarct size, improved cardiac function, suppressed myocardial apoptosis, oxidative damage and I/R-induced ER stress; Reduced cell apoptosis, oxidative stress and ER stress of cultured cardiomyocytes	Activation of JAK2/STAT3 signaling pathway and attenuation of ER stress-induced apoptosis	Zhao et al., 2016b
	Berberine	*In vivo*	Attenuation of I/R-induced incidence of ventricular arrhythmia and amelioration of myocardial histological changes	Inhibited activation of PI3K/AKT signaling and subsequent reduced expression of IL-6, IL-1β and TNF-α	Zhu and Li, 2016

## Cardioprotective activities of Rhizoma Coptidis

### Anti-atherosclerotic effect

Atherosclerosis is one of the most important causative factors of certain CVDs such as myocardial infarction (MI), heart failure, stroke and claudication (Frostegård, 2013). Atherosclerosis refers to a specific form of arteriosclerosis in which an artery-wall thickens due to invasion and accumulation of lipid laden white blood cells (foam cells) and proliferation of intimal-smooth-muscle cell forming a fibrofatty plaque (Lucas and Greaves, 2001). There have also been studies supporting the anti-atherosclerotic effects of RC. For example, a study on the anti-atherosclerotic potential of Ger-Gen-Chyn-Lian-Tang (a traditional mixture which contains berberine as one of its major active components) has demonstrated reduced atherosclerotic lesions and collagen expression within the atheroma plaques in mice. In addition, *in vitro* work has also shown a reduction in migration of vascular smooth muscle cells, which is significant as pathological migration of these cells represents a key step in the pathogenesis of atherosclerosis (Ho et al., 2012).

Reactive oxygen species (ROS) play a crucial role in the development and progression of atherosclerotic lesions because the formations of lesions are related to several ROS-regulated events. It was found that uncoupling protein 2 (UCP-2), the mitochondrial inner membrane protein can provide antioxidant mediated defense by negatively regulating ROS production (Moukdar et al., 2009). Utilizing a mouse model, Wang et al. (2011) reported that chronic administration of berberine resulted in significant reduction of aortic lesions, diminished oxidative stress and lowered expression of adhesion molecules in the aorta; this anti-atherogenic activity was due to AMPK-dependent ROS suppression through enhanced expression of UCP-2. Oxidative modification of LDL is also believed to be one of the main risk factors leading to atherosclerosis (Itabe, 2009); berberine—one of the major bioactive components of RC—has been reported to inhibit oxidation of LDL by virtue of its significant antioxidant activity (Hsieh et al., 2007). Berberine also appears to protect against oxidized-LDL induced apoptosis as demonstrated by reduced levels of mitochondrial cytochrome *C*, cleaved apoptotic effectors, caspase 3 and poly(ADP-ribose) polymerase in human umbilical vein endothelial cells treated with this compound (Hsieh et al., 2007). Magnoflorine, another alkaloid isolated from RC extract also possesses similarly beneficial effects; an effective antioxidant, it prevents the oxidation of various forms of LDL, including native, glycated and glycoxidated LDL (Hung et al., 2007a). Aside from this, magnoflorine also significantly inhibits Cu^2+^ and thermo-labile radical initiator (AAPH)-induced lipid peroxidation of high density lipoprotein (HDL); thus resulting in HDL retaining its ability to protect LDL from oxidation (Hung et al., 2007b).

Chronic inflammatory processes are a key additional factor leading to the development of atherosclerotic lesions in the vessel wall (Remppis et al., 2010). Hence, substances with anti-inflammatory property may have the potential to prevent atherosclerosis. Remppis et al. (2010) studied the effect of RC extract and berberine on lipopolysaccharide (LPS)-induced inflammatory activity in a murine macrophage cell line. The results of this study demonstrated that RC extract and berberine inhibit the production of monocyte chemoattractant protein 1 (MCP-1/CCL2) in macrophages, likely via inhibition of the transcription factors activator protein 1 (AP-1) and nuclear factor-kappaB (NFκB). Coptisine, another of the bioactive compounds in RC, also possesses demonstrable anti-inflammatory property. It resulted in decreased nitric oxide (NO) production in LPS-stimulated macrophages via inhibited protein and mRNA expression of inducible nitro oxide synthase (iNOS); additionally, the expression of pro-inflammatory cytokines interleukin (IL)-1β and IL-6 was inhibited at transcriptional level. The proposed underlying mechanism for these observations was blockade of NFκB, mitogen-activated protein kinases (MAPK), and phosphoinositide-3-kinase (PI3K)/Akt activation, which are the intracellular inflammation signaling pathways in macrophages (Supriady et al., 2015; Wu et al., 2016).

The accumulation of lipid-laden foam cells is also an important step in the progression of atherosclerosis (Chi et al., 2014). The authors demonstrated that berberine effectively inhibited oxidized-LDL-induced foam cell formation through the activation of adenosine 5′-monophosphate-activated protein kinase (AMPK)-sirtuin 1-peroxisome proliferator-activated receptor gamma (PPAR-γ) pathway as well as through reduced uptake of oxidized LDL by foam cells. Furthermore, the same study showed that combination therapy of berberine and atorvastatin (a well-established anti-atherosclerotic drug) was more effective in preventing atherosclerotic processes compared to administration of atorvastatin alone.

Clinically, the major concern when artherosclerosis is present is the occurrence of plaque disruption which may then lead to unstable angina, or an outright MI. Plaque progression and destabilization is due to the over-expression of matrix metalloproteinases-9 (MMP-9) and extracellular matrix metalloproteinase inducer (EMMPRIN) in monocytes/macrophages (Cao et al., 2014). Berberine inhibits the up-regulation of MMP-9 and EMMPRIN in phorbol 12-myristate 13-acetate-induced macrophages by suppressing the activation of p38 pathway, showing its ability to stabilize atherosclerotic plaque (Huang et al., 2011).

RC seems to have multiple active components that target varying stages of the development of atherosclerosis through a variety of pathways, thus making it an effective anti-atherosclerotic agent.

### Lipid-lowering effect

Hyperlipidemia or dyslipidemia, which is characterized by increased plasma triglyceride concentration and lowered HDL cholesterol, is one of the major risk factor contributing to the progression of CVDs such as CHD and peripheral artery disease. Hence, maintenance of total cholesterol (TC) level within the normal range can be an effective strategy to lower the risk of cardiovascular events (Zou et al., 2016). The liver is the main site of cholesterol homeostasis maintenance, which involves several distinct processes including biosynthesis via the activity of 3-hydroxy-3-methylglutaryl coenzyme A reductase (HMGCR), uptake by low density lipoprotein receptors (LDLR), release of lipoprotein in the blood, storage, degradation and conversion into bile acids (Trapani et al., 2012). RC possesses lipid-lowering effects as shown by the lowered cholesterol levels in different animal models treated with RC extract (Yokozawa et al., 2003; Cao et al., 2012; Kou et al., 2016). Studies have indicated that the five main bioactive compounds in RC (berberine, coptisine, palmatine, epiberberine, and jatrorrhizine) exert their lipid-lowering effects through distinct mechanisms. Berberine's hypolipidemic effect was the result of up-regulation of LDLR mRNA and protein expression (Zhou et al., 2014). Coptisine treatment also resulted in increased expression of LDLR, and additionally was associated with up-regulated expression of cholesterol 7-alpha-hydroxylase (CYP7A1) and down-regulated expression of HMGCR (Chen et al., 2015; He et al., 2015b). In addition to up-regulated expression of LDLR and CYP7A1, treatment with palmatine and epiberberine also caused down-regulation of apical sodium-dependent bile salt transporter (ASBT) mRNA and protein expression, as well as enhanced fecal excretion of TC and total bile acids (Ning et al., 2015; Zou et al., 2016). Jatrorrhizine has been linked to up-regulated mRNA and protein expression of LDLR and CYP7A1 but it has no effect on the expression of ASBT and HMGCR (Wu et al., 2014a). Comparative studies have been done on the lipid-lowering effect of individual alkaloids and among those tested, coptisine was reported to have the highest lipid-lowering effect (Wu et al., 2014c). More recently, research involving combined treatment with the alkaloids in RC extract has also been carried out. While each of the compounds administered in isolation did demonstrate lipid-lowering effect, the administration of a combination of these alkaloids resulted in synergistic effects, with combination therapy resulting in a significantly greater lipid-lowering effect compared to treatment with a single alkaloid. The main mechanisms associated with this effect are thought to be the retardation of cholesterol synthesis and accelerated clearance of cholesterol (He et al., 2015a; Kou et al., 2016).

Type 2 diabetes is strongly associated with significant morbidity and mortality due to cardiovascular complications. This is in part due to the impaired utilization of carbohydrate that is part of the metabolic profile of diabetes, which results in accelerated lipolysis and thus hyperlipidemia or dyslipidemia (Vijayaraghavan, 2010; Ma et al., 2016). RC has been shown to have the ability to attenuate the impaired lipid metabolism associated with diabetes. JiuHuangLian (RC steamed with rice wine), a traditional Chinese medicine preparation has been reported to restore the disordered lipid metabolism of type 2 diabetic rats, by decreasing the TC, total glyceride and free fatty acid levels (Li et al., 2013). The anti-hyperlipidemic effect of the alkaloids isolated from RC have been further tested on diabetic mice. Palmatine and jatrorrhizine were shown to decrease the concentrations of serum TC and triglyceride; while elevated HDL cholesterol levels were found in diabetic mice administered with palmatine, jatrorrhizine and berberine (Ma et al., 2016). In another study, there were decreased serum TC, triglyceride, LDL cholesterol and increased HDL cholesterol in diabetic rats treated with berberine (Tang et al., 2006), showing the ability of RC to attenuate the impaired lipid metabolism in diabetic conditions.

In short, RC has remarkable lipid-lowering effects, mainly through inhibition of cholesterol synthesis, in combination with increased usage and excretion of cholesterol.

### Anti-obesity effect

Obesity has become a global epidemic and is strongly associated with occurrence of CVDs including heart failure, CHD, sudden cardiac death and atrial fibrillation (Lavie et al., 2009). As the condition is characterized by accumulation of extra body fat into adipocytes, the typical feature of obesity is an increase in the number and size of adipocytes. The etiology of obesity is rooted in the process of differentiation of preadipocytes into adipocytes, known as adipogenesis (Kwak et al., 2013; Choi et al., 2014). The key transcription regulator genes involved in adipogenesis are PPAR-γ and CCAAT/enhancer binding protein alpha (C/EBP-α). The inhibition of these two transcription factors could represent an effective method of inhibiting adipogenesis (Choi et al., 2014). RC has potential as an anti-obesity agent as the extract of RC as well as its isolated alkaloids have demonstrated the ability to inhibit cellular triglyceride accumulation in 3T3-L1 adipocytes. Significant down-regulation of expression of PPAR-γ and C/EBP-α by the alkaloids was also demonstrated, which is consistent with the reported mechanism for the anti-adipogenic activity of berberine (Huang et al., 2006).

The anti-obesity potential of RC has also been demonstrated in *in vivo* studies. The administration of RC and berberine to mice fed a high fat diet has resulted in significantly lower body and visceral adipose weight. Interestingly, this effect has been related to the antimicrobial activities of both RC and berberine; with reduced fecal microbes resulting in decreased degradation of dietary polysaccharides, lower calorie intake and *de novo* lipogenesis. This was followed by activation of fasting-induced adipose factor (Fiaf) protein and related gene expressions of mitochondrial energy metabolism such as AMPK, PGC1α, UCP2, CPT1α, and Hadhb in visceral adipose tissue (Xie et al., 2011). Recently, the gut microbiota of obese mice treated with RC alkaloids has been further studied, demonstrating an increased abundance of microbial species negatively associated with obesity namely *Sporobacter termitidis, Alcaligenes faecalis*, and *Akkermansia muciniphila*, and a decreased abundance of the microbial species positively associated with obesity namely *Escherichia coli, Desulfovibrio C21 _c20* and *Parabacteroides distasonis*. This indicates the ability of RC to prevent the development of obesity by restoring the balance of disrupted gut microflora associated with the obese condition (He et al., 2016). RC appears to be effective in preventing obesity as it is able to inhibit adipogenesis and modulate the gut microflora that plays important role for the development of obesity.

### Anti-hepatic steatosis effect

Nonalcoholic fatty liver disease (NAFLD)—a common liver disease with a disease spectrum ranging from simple steatosis to steatohepatitis—is regarded as the hepatic manifestation of metabolic syndrome (Liu and Lu, 2014). Recently, hepatic steatosis has been identified as one of the associated risk factors closely related to the morbidity and mortality of CVDs. It has been reported that patients with NAFLD have higher risk of getting CHD compared to the general population (Pisto et al., 2014). There are several possible mechanisms by which hepatic steatosis may contribute to the risk of CVDs including oxidative stress, inflammation, dyslipidemia, ischemia reperfusion injury, visceral fat, low adiponectin, ectopic adipose tissue distribution, endothelial dysfunction and postprandial dyslipidemia (Liu and Lu, 2014). In spite of the many advances in modern medicine, there is as yet no effective treatment of NAFLD (Huang et al., 2013). Herbal medicine has been an area of interest for scientists seeking new modalities of treatment of hepatic steatosis due to the low incidence of side effects and promising therapeutic benefits (Xiao et al., 2013; Tan et al., 2016). For example, Jiao Tai Wan, a classic traditional Chinese prescription consisting of RC and Cortex Cinamomi was able to inhibit hepatic lipid accumulation in diabetic rats and humans, due to down-regulation of lipogenic gene expression and inhibition of lipogenesis in liver tissue (Huang et al., 2013). Sam-Hwang-Sa-Sim-Tang, a Korean traditional preparation which contains RC, Scutellaria radix and Rhei rhizoma also exhibited protective effect against hepatic steatosis by inhibiting the expression of hepatic molecules that modulate cholesterol metabolism (Ahn et al., 2013). While RC extract clearly does have significant potential as an anti-hepatic steatosis agent, there has been interest in identifying which of the extract's active components exert the greatest anti-hepatic steatosis effect. Using a free fatty-acid induced hepatic steatosis HepG2 cell assay, Fan et al. (2013) demonstrated that berberine and coptisine are the alkaloids with strongest inhibitory effect on triglyceride accumulation. This is consistent with the findings of other studies that proposed berberine as the effective compound against hepatic steatosis (Yuan et al., 2015; Guo et al., 2016; Zhang et al., 2016). In short, RC possesses anti-hepatic steatosis effect which is related to its ability to inhibit hepatic lipogenesis.

### Protection from myocardial ischemia-reperfusion injury

Coronary artery disease resulting in compromised oxygen supply to the myocardium is a common cause of morbidity and mortality. Medical intervention now makes it possible to achieve rapid restoration of blood flow to the ischemic myocardium following an acute event, which should produce better clinical outcomes as prompt restoration of blood circulation helps to prevent further tissue injury. However, the restoration of blood flow or reperfusion, will also cause injury, which is known as myocardial ischemia reperfusion injury (I/R) (Yu et al., 2015). Apoptosis is an important cellular mechanism resulting in I/R injury, with oxidative stress representing one of the major contributing factors promoting apoptosis. The excessive production of ROS which are mainly produced by mitochondria will initiate apoptotic signaling (Chan et al., 2012, 2015). Hence, reducing oxidative stress may help inhibit apoptosis thus potentially providing an effective strategy to attenuate I/R injury (Fan et al., 2012). Traditional Chinese herbal treatment have been commonly used to treat MI for hundreds of years and recently, it has also been reported to exert protective effect against myocardial I/R injury (Liu et al., 2013; Li et al., 2014; Zhao et al., 2016a).

One example of the traditional Chinese medications is San-Huang-Xie-Xin-Tang (SHXT), which consists of three herbs: RC, *Scutellariae radix* and *Rhei rhizome*. Liou et al. (2011) showed that pre-treatment with SHXT was associated with significantly lower arrhythmia scores, reduced mortality rates and reduced levels of cardiac enzymes such as creatine kinase (CK), lactate dehydrogenase (LDH) and troponin I in rats with myocardial I/R injury. Additionally, there was reduced infarct size and decreased apoptosis in myocytes, partly due to modulation of endothelial nitric oxide synthase (eNOS) and MAPK pathways. While it is commonly used as a component of herbal mixtures, the extract of RC alone has been shown to possess cardioprotective effect against I/R damage. Administration of crude extract of RC for 7 days preceding acute myocardial injury in rats was associated with reduced myocardial injury as evidenced by decreased ST-T elevation on electrocardiogram (ECG) and biochemically by decreased levels of CK and LDH. Other beneficial effects were reduction in oxidative stress reflected by reduced levels of malondiealdehyde (MDA) and increased activity of superoxide dismutase (SOD) (Liu et al., 2010).

Several active compounds identified in RC have been suggested as possible candidates accounting for the cardioprotective activities against I/R injury as demonstrated. One of the compounds that appears to have cardioprotective activity is berberine. Although the cardioprotective activity of berberine is well recognized, the exact mechanism by which it protects from I/R injury remains unknown. Chang et al. (2012) found that pretreatment with berberine decreased infarct size and reduced the incidence as well as duration of arrhythmias in rats with I/R injury with demonstrable difference in AMPK activity in both non-ischemic areas (NIA) and myocardial areas at risk (AAR). Up-regulation of AMPK in the NIA helped to enhance cardiac function through increased uptake of glucose, glycolysis and oxidation of fatty acid whereas down-regulation of AMPK in the areas at risk helped to protect the cardiac myocytes from apoptosis. This protective effect of berberine was later specifically demonstrated in the CVD prone diabetic heart, as demonstrated by the regulation of myocardial energy metabolism by berberine during I/R injury (Chang et al., 2016). In myocardial I/R, there is always excessive autophagy which will lead to expansive degradation of cytosolic proteins and organelles, resulting in collapse of cellular functions. Huang et al. (2015) have reported the beneficial effect of berberine in lowering the cellular autophagy level in berberine-treated myocytes exposed to I/R injury which were found to have reduced expression of autophagy-related proteins, which then led to suppression of autophagy activation. Berberine's cardioprotective action may also be associated with its anti-apoptotic action. Based on both *in vitro* and *in vivo* results, berberine decreases I/R-induced myocardial apoptosis, as a result of modulation of the Notch1/ Hairy and enhancer of split 1(Hes1)- Phosphatase and tensin homolog deleted on chromosome 10 (PTEN)/Akt signaling pathway (Yu et al., 2015). In a diabetic rat model, berberine exerted anti-apoptotic activity through activation of AMPK and PI3K-Akt-eNOS signaling (Chen et al., 2014).

Mitochondrial dysfunction is an additional factor believed to be responsible for myocardial I/R injury. Treatment with berberine has been shown to improve mitochondrial dysfunction, as demonstrated by improved mitochondrial membrane potential, increased mitochondrial complex I activity and reduced release of cytochrome *C* from the mitochondria (Wang et al., 2015). Endoplasmic reticulum (ER) stress is also known to play an important part during I/R injury. Unfolded protein response (UPR) is initiated in the myocardium after I/R and the continuous UPR causes upregulation of proapoptotic proteins. Consequently, cellular apoptosis occurs, resulting in myocardial I/R injury (Wu et al., 2014b). The role of berberine in modulation of ER stress level during myocardial I/R injury has also been described. Pretreatment with berberine has resulted in suppressed myocardial I/R induced ER stress due to the activation of Janus kinase 2/signal transducer and activator of transcription 3 (JAK2/STAT3) signaling (Zhao et al., 2016b) An additional mechanism by which berberine is able to ameliorate myocardial I/R injury is via repression of the inflammatory response. Lower levels of inflammatory markers, namely the cytokines tumor necrosis factor (TNF)-α, IL-6 and IL-β were found in rats pre-treated with berberine prior to induction of myocardial ischemia; this was believed to be mediated by downregulation of PI3K/AKT signaling thus preventing myocardial I/R injury (Zhu and Li, 2016).

Coptisine is one of the other compounds in RC that might have a role in prevention of I/R injury. In a rat model, administration of coptisine alleviated I/R-induced arrhythmias and attenuated the reduction in ejection fraction as well as fractional shortening on echocardiography. Treatment with coptisine was also associated with reduced infarct size, suppressed myocardial apoptosis and reduced proinflammatory cytokines. Based on the reduced expression of Rho, Rho-kinase 1 (ROCK1), ROCK2 and attenuation of myosin phosphatase targeting subunit-1 phosphorylation, it was speculated that inhibition of the Rho/ROCK pathway was likely to be coptisine's mechanism of cardioprotection (Guo et al., 2013).

Palmatine, another component contained in RC extract, is also recognized as a potential cardioprotective agent. The administration of palmatine to rats prior to myocardial I/R injury was associated with reduced I/R–induced myocardial dysfunction as evidenced by inhibition of the expected increase in LDH, CK, and MDA, as well as significant reduction in cyclooxygenase-2 (COX-2) and inducible nitric oxide synthase (iNOS) expression. Interestingly, *in vitro* studies using human aortic endothelial cells resulted in increased heme-oxygenase-1 induction, indicating the strong antioxidant and anti-inflammatory action of palmatine (Kim et al., 2009). Based on the studies, RC is a potential protective agent against myocardial I/R injury through its active compounds which exert different protection actions including regulation of cellular energy metabolism, anti-apoptosis effect, protection against mitochondrial dysfunction and ER stress as well as antioxidant and anti-inflammatory action.

## Conclusion

In conclusion, results of recent studies appear to support for the usage of RC for treatment of CVDs and related conditions. They have provided scientific evidence that RC's use in traditional Chinese medicine for the last 2000 years was likely to have truly resulted in desirable effects on CVDs via its effects on the various risk factors and biochemical pathways involved in CVDs pathogenesis. RC appears to have tremendous potential as a cardioprotective agent given its ability to improve a large number of parameters associated with increased risk of CVDs including anti-atherosclerotic effect, lipid-lowering effect, anti-obesity effect and anti-hepatic steatosis effect. Aside from reducing incidence, it also reduces the damage caused by CVDs as it confers protection from myocardial I/R injury. These properties are mainly attributed to its bioactive compounds: berberine, coptisine, palmatine, epiberberine, jatrorrhizine and magnoflorine. However, knowledge regarding the underlying mechanisms of action of these compounds is still limited. More research investigating the exact mechanisms of RC's cardioprotective activities is needed. This is to fully exploit this traditional remedy's potential to contribute to the development of new cardioprotective agents as this could be a new opportunity to reduce global prevalence of CVDs.

## Author contributions

HT and BG contributed to the literature database search, data collection, data extraction, and writing of the manuscript. PP, SS, AD, TM, KC, LL, and BG contributed vital insight and proofread on the writing. The research topic was conceptualized by BG.

### Conflict of interest statement

The authors declare that the research was conducted in the absence of any commercial or financial relationships that could be construed as a potential conflict of interest.

## Abbreviations

ACC: acetyl coenzyme A carboxylase
ADP: adenosine monophosphate
AMPK: adenosine 5′-monophosphate (AMP)-activated protein kinase
AP-1: activator protein 1
ASBT: apical sodium-dependent bile salt transporter
ATP: adenosine triphosphate
Bax: Bcl-2-associated X protein
Bcl-2: B cell lymphoma 2
BNIP3: BCL2/adenovirus E1B 19kDa interacting protein 3
C/EBP-α: CCAAT/enhancer-binding protein- α
CK: creatine kinase
COX-2: cyclooxygenase-2
CPT1α: carnitine palmitoyl transferase 1-alpha
cTn1: cardiac troponin I
CYP7A1: cholesterol 7-alpha-hydroxylase
EMMPRIN: extracellular matrix metalloproteinase inducer
eNOS: *endothelial nitric oxide synthase*
FAS: fatty acid synthase
FXR: farnesoid X receptor
GSK3β: glycogen synthase kinase 3β
HDL: high density lipoprotein
HDL-c: high density lipoprotein cholesterol
Hes1: Hairy and enhancer of split 1
HMG-CoA: 3-hydroxy-3methylglutary-CoA
HMGCR: 3-Hydroxy-3-methylglutaryl CoA reductase
HO-1: heme-oxygenase-1
HUVECs: human umbilical vein endothelial cells
IL: Interleukin
I/R: ischemia/reperfusion
iNOS: inducible nitric oxide synthase
JAK: Janus kinase
JNK: c-Jun N-terminal kinase
LDH: lactate dehydrogenase
LDL: low density lipoprotein
LDL-c: low density lipoprotein cholesterol
LDLR: low-density lipoprotein receptor
LPS: Lipopolysaccharide
LXR: liver X receptor
MAPK: p38 mitogen-activated protein kinase
MCP-1: monocyte chemoattractant protein-1
MDA: malondialdehyde
MMP: matrix metalloproteinase
mTORC2: mammalian target of rapamycin complex 1
NFκB: Nuclear factor-kappaB
Ox-LDL: oxidized low density lipoprotein
pACC: phosphorylated-ACC
PGC1α: peroxisome proliferator-activated receptor gamma coactivator 1-alpha
PI3K: phosphoinositide 3-kinase
PMA: phorbol 12-myristate 13-acetate
PPAR: proliferator-activated receptor
PTEN: Phosphatase and tensin homolog deleted on chromosome 10
ROCK: Rho-kinase
ROS: reactive oxygen species
SIRT1: Sirtuin 1
SOD: superoxide dismutase
SREBP2: Sterol regulatory element binding transcription factor 2
STAT3: signal transducer and activator of transcription 3
TBA: total bile acids
TBARS: thiobarbituric acid reactive substances
TC: total cholesterol
TG: triglyceride
TGR5: G-protein coupled bile acid receptor
TLR4: toll-like receptor 4
TNF: tumor necrosis factor
TXNIP: thioredoxin-interacting protein
UCP 2: uncoupling protein 2
VMSCs: vascular smooth muscle cells.
